# Cleft Palate and Artificial Velum

**Published:** 1888-06

**Authors:** J. A. Biggs


ARTICLE IX.

### CLEFT PALATE AND ARTIFICIAL VELUM.

BY MR. J. A. BIGGS, L. D. S. GLAS.

The case I bring before you on this occasion is one of congenital cleft palate. I may, in passing, tell you that staphyloraphy had been performed on this patient on two successive occasions without any benefit to her whatever. The young lady, æt. 21, is the daughter of a physician of about thirty years' standing in Glasgow. There is no history of any similar tendency in her family. Staphyloraphy having proved a failure on two occasions on which it was performed, you can readily realize that there was a considerable dubiety in the minds of her parents as to the advisability of again calling in the aid of science; but having reached maturity, with this deformity preventing her from making herself intelligible to any but her immediate relations, they ultimatively came to the determination to make one more venture on her behalf, to which determination they were aided by the late Dr. Hugh Miller, who advised that she be placed in my hands, with the view of making her an artificial velum.

On examination, I found she had a small contracted superior maxilla, containing nothing worth retaining except the two wisdom teeth; the first bicuspid on the right, and a remnant of the corresponding tooth on the left. The wisdom teeth were small, malformed, almost useless things, and the ravages made upon the left bicuspid by decay rendered it difficult to make much use of it, so that the upper right was all that could be relied on for the purpose of retaining a denture in the mouth. I did not remove a single root, but cut the crown level with the gum; then I made a ferrule to fit the decayed bicuspid, and, after thoroughly filling the root, forced the ferrule up over it, up to and below the gum, and then filled it up with amalgam, and thus made it a good serviceable anchor. I also enlarged the nerve canal of the upper central incisor for the purpose of introducing a pivot, which would thoroughly prevent any unsteadiness anterio posteriorly in the denture. The fissure ran from the pharyngeal walls forward to about one inch and an eighth from the alveolar borders, or opposite the second bicuspid teeth, at which part the width of the fissure was three-eighths of an inch, and gradually widening towards the divided uvula, the two halves of which were about five-eighths of an inch apart; and behind, from the tensor palati of the left to that of the right, was about an inch and a-half, and from the pharyngeal wall to the uvula, about three-quarters of an inch. The anterior and posterior nares were in full view; and also the vomer and the inferior and superior turbinated bones.

I took an ordinary impression tray and some modelling composition, and procured an impression of the hard plate, reaching back as far as possible, but not allowing the material to flow into the fissure. I then struck up a very light impression tray from block tin, and soldered a handle to it; then added a tray of gutta-percha to its extremity. I then filled with plaster of Paris, placed a wire in the root of the central, and then introduced the plaster. I may here mention that the patient is very delicate, and owing to great sensitiveness, retching was easily provoked. I had to administer stimulants to keep her altogether, and also to paint the fauces, &c., with dilute phenol sodique, to abate the sensitiveness and retching.

When this plaster-impression was set, I removed it, then cut a hole in the tray, containing the plaster-impression; and through this I introduced an India-rubber tube, soaped the surface, and returned it to the mouth. An assistant held it in position there. I then pushed the nozzle of a syringe into the end of the tube, and withdrew the plunger. Another assistant had plaster mixed with potass alum ready. This was poured into the syringe, the plunger was replaced, and the plaster injected into the fissures; then I withdrew the first part of the impression; and with good strong tweezers pushed the other part backwards as if down the throat.

Both halves of the impression were then placed in situation and then cast, and, to facilitate the getting at either side, the model was also taken in two parts. The model was then stearined, and the fissure filled, and an impression in sand taken, and thus Babbit metal models secured, on which a gold plate was swaged; bands fitted to the bicuspids, and light wires encircling the wisdom teeth, and a wire soldered into the plate, to fit into the left central incisor.

The six front teeth were fitted and backed, and at this stage the whole was fitted into the mouth, and found most satisfactory; they were then soldered and tried upon the model but the plate, from some cause or other, was very badly warped Probably the assistant had used too heavy an investment, that is, an excess of material, causing a twist that was retained by the solder. This was somewhat disheartening, because, with the teeth bands and pivot, it became a diffioult matter to recover the fit. There, however, were only two ways for it—either to make a new one or to re-strike that one up again. I chose the latter plan, and proceeded thus:—I placed an iron wire in the pivot root, made up a mixture of asbestos, fireclay, and plaster of Paris, soaped the model, and took an impression, dried, and cast in Babbitt metal.

The pin was easily withdrawn, having been coated with whiting. The front part of the model was covered with fireclay, asbestos, and plaster of Paris, dried, and the lead poured over. This formed a chamber over the teeth and bands of the plate. So the plate, with teeth bands, pivot included, was restruck, and the fit restored. I have gone into detail, which may seem tedious to some; but I thought it might be interesting to others, and also instructive to those who did not know of this method, and therefore useful to them if a like contingency should be met with in their practice.

The bicuspids and molars of the lower jaw were all so irretrievably lost that for the present no cognisance was taken of them, and vulcanite blocks were rivetted on the plate as substitutes for the bicuspids and molars.

Holes were now punched in the palate of the gold plate, and a small piece of gold fitted to it, to which two gold screws were soldered, having small nuts working freely upon them. The object of this was to secure a hinge, also of gold, to which was attached the artificial velum. There is a small portion of the fissure covered by the anterior part of the hinge. This was mounted with rubber to act as an obturator. To the posterior portion of the hinge was adapted the artificial velum. At first my intention was to make this part in soft rubber, but further consideration made me determine to construct it in hard rubber. I believe that far better results may be obtained from soft rubber than is usual if it be packed in a tin mould, and vulcanized in dry heat, as obtained by the new-mode heater. There is, as on cast metal, an impermeable skin compared with vulcanite cooked in steam. But the best cooked soft rubber will not wear so well as plain hard rubber, and will require renewing from time to time. For that reason I determined to make it of hard rubber, which I proceeded to do thus:—I attached the hinge to the plate, placed it on the model softened in boiling water some gutta-percha, and modelled up the velum. This I cooled and tried in the mouth, and, after some slight adjusting, I was satisfied with its position and its adaptation. Owing to the difficulty of packing it, it was flasked, so that the side parts were capable of being removed like cores, but it was found unnecessary to make use of them practically.

When the velum was finished, it was attached to the plate by the screws and nuts, and tried in the mouth, and the necessary pitch obtained by the rising of the stop pin. This, as you observe, is a contrivance for taking the weight of the case off the muscles when at rest. You will observe that part of it lies above the natural velum and part below; so that when the uvula is raised or depressed, the artificial velum is carried along with it, and thus fulfilling physiologically the purpose for which it was constructed.

I need not point out to you the great difficulties to be overcome by the patient, even after all that is possible has been done. A patient with a congenital cleft of the palate has never been able to pronounce distinctly any words, and but few sounds. Therefore, after the introduction of such an instrument, it requires an actual course of training to teach them to pronounce sounds they have never learned to do before, and this patient is no exception to the rule. There is, however, a marked improvement in her capacity to utter sounds, and even already she has learned to repeat sounds she could not imitate at all before. She had only worn the plate and artificial velum a fortnight, and it took the best part of that time to familiarize her with it.—*London Dental Record.*

---

